# Is There a Shortage in the Supply of Nurses?

**Published:** 1916-12-30

**Authors:** E. W. Morris

**Affiliations:** House Governor. London Hospital, Whitechapel, E.


### THE EDITOR'S LETTER-BOX.

### Is there a Shortage in the Supply of Nurses?

*To the Editor of* The Hospital.

Sir,—There has been a good deal in the newspapers lately as to the shortage in the supply of nurses, and in more than one instance I note that it is stated that one reason, at any rate, for the shortage in the supply of nurses is the matter of under-pay. It may be of interest to ladies who intend to take up nursing to know what are the payments made to probationers, nurses, and sisters. I can only give our own figures exactly, but I suppose all hospitals give salaries somewhat like these :—No fee is taken for training. A nurse in her first year of training is paid £17; in her second year, £24; in the third year of her engagement she becomes a staff nurse, and is paid £30. When made a ward sister her pay is from £40 to £50. There are several special appointments open to sisters, six of which at this hospital rise to £100. She gets an additional £5 to her salary for every six years she serves. She gets a further £5 in addition to her salary if she, at her own expense, obtained a Central Midwives Board certificate. At the age of forty-five, and after eighteen years' service, she can retire with full pay for life, and is not prohibited from holding any other appointment she may wish to after leaving us. The daily, monthly, and annual leave also is generous, but I need not go into that now.—Yours faithfully,

E. W. Morris, House Governor.

London Hospital, Whitechapel, E., December 22, 1916.

